# Editorial: Armed conflicts; implications, dynamics and impacts on public health care services

**DOI:** 10.3389/fpubh.2022.1008883

**Published:** 2022-09-05

**Authors:** Mohamed A. Daw

**Affiliations:** Department of Medical Microbiology & Immunology, Faculty of Medicine, University of Tripoli, Tripoli, Libya

**Keywords:** armed conflict, impacts, public healthcare, dynamics, implication

Armed conflicts create major complications for the affected nations and cannot be easily accounted or determined. These may include immediate effects manifested in high rates of mortality, injury, and the predicament of millions of refugees and internally displaced peoples, as well as major destruction of healthcare services and dissemination and emergence of new microbial diseases. Armed conflicts have increased tremendously and spread widely tearing down the moral content of modern civilization (1). They are no longer confined to developing countries such as the Middle East and North Africa (MENA) region as in Libya, Syria, Yemen, and Iraq, but go beyond to reach the heart of Europe as illustrated by the current situation in Ukraine.

This Research Topic explores the implications, dynamics, and the impacts of armed conflicts on healthcare services. To include but not limited to destruction of healthcare services and changes in the transmission dynamics of microbial diseases during and after the conflicts. Given this context, there are four core arguments that we advance in this Research Topic:

The first implication of armed conflicts which was profound among the affected countries was the destruction of social and identity contents of the affected nation. This was not limited to certain sectors, such as healthcare services, but it also reached other sectors including social, education, economic services, and even emotional and ritual thinking (2). The destruction of healthcare and education systems is one of the most devastating impacts of armed conflicts. This is clearly evident in Libya which was considered to be on the top of the developing countries on healthcare and social services according to the human development index. Nowadays, the Libyan healthcare system has collapsed and citizens are not able to meet basic needs or access emergency services (3).

The second core impact is the effects of the armed conflict on the dissemination and transmission-dynamics of infectious disease. This is clearly evident from the impact of the armed conflict on the epidemiological situation of the COVID-19 pandemic in Libya, Syria, and Yemen. The armed conflict in such countries hindered the efforts to fight the pandemic and acted as a catalyst for its spread resulting in more serious consequences (4). In prolonged conflicts as in occupied Palestine, the situation is more serious, particularly for immunocompromised patients such as cancer patients whose access to services is interrupted during the spread of COVID-19 pandemic (Sabateen et al.).

Furthermore, the impact of the armed conflict on the prevalence and transmission-dynamics of HIV/AIDS is clearly evident in Libya and Ukraine. The transmission of HIV-1 increased and the migration of HIV strains represents an enormous monitoring challenge in these countries making it difficult to control. In Libya the armed conflict has complicated the spread of HIV and has resulted in the spreading of new HIV strains and the buildup of HIV cases in western and middle regions which did not exist before the armed conflict (Daw et al.).

The third concern is the intervention policy to combat the consequences of the armed conflict, particularly at the emergence of pandemics during the armed conflict. This necessitates local authorities and humanitarian organizations (e.g., red crescent/cross) to protect people's health by developing and evaluating the ongoing short- and long-run policies. However, shelter hospitals were developed for the isolation and treatment of patients with infectious diseases during the conflict (Hu et al.).

The fourth dilemma is how to deal with consequences of the armed conflict, particularly the migrated and displaced people. In the recent Ukraine conflict, over 12 million people particularly children, women, elderly, and people with special needs migrated to Europe as a result of the conflict. This emphasizes the need to provide medical resources and accommodation. Hence, the governmental and non-governmental organizations, foundations, and medical network should intervene to provide the essential needs of medical, social, and economic care for the immigrants (Fatyga et al.).

Injury and intentional disabilities are the two major complications associated with armed conflict that inflicted heavy burdens on society that may persist for a long time (5). However, disabilities caused by blasts or gun shots could result in more deaths and leave devastating impacts upon the physical, social, and vocational wellbeing of patients (Zhang et al.).

## Author contributions

MD designed, wrote, and completed the manuscript

## Conflict of interest

The author declares that the research was conducted in the absence of any commercial or financial relationships that could be construed as a potential conflict of interest.

## Publisher's note

All claims expressed in this article are solely those of the authors and do not necessarily represent those of their affiliated organizations, or those of the publisher, the editors and the reviewers. Any product that may be evaluated in this article, or claim that may be made by its manufacturer, is not guaranteed or endorsed by the publisher.

## References

[1] Khorram-Manesh A Burkle FM Goniewicz K Robinson Y. Estimating the number of civilian casualties in modern armed conflicts-a systematic review. Front Public Health. (2021) 9:765261. 10.3389/fpubh.2021.765261 34778192 PMC8581199

[2] Daw MA. Libyan healthcare system during the armed conflict: challenges and restoration. Afr J Emerg Med. (2017) 7:47–50. 10.1016/j.afjem.2017.04.010 30456107 PMC6234156

[3] Libya: UN HDI Country Profile, Posted Posted by Alexandra Valiente on March 5, 2011 [Internet] (2014). Available online at: https://libyadiary.wordpress.com/2011/03/05/libya-un-hdi-country-profile/ (accessed December 12, 2016).

[4] Daw MA. The impact of armed conflict on the epidemiological situation of COVID-19 in Libya, Syria and Yemen. Front Publ Health. (2021) 9:667364. 10.3389/fpubh.2021.667364 34178925 PMC8226094

[5] Daw MA El-Bouzedi AH Dau AA. Trends and patterns of deaths, injuries and intentional disabilities within the Libyan armed conflict: 2012-2017. PLoS ONE. (2019) 14:e0216061. 10.1371/journal.pone.0216061 31075119 PMC6510427

